# Hyper-Cross-Linked Porous Polymer Featuring B–N Covalent Bonds (HCP-BNs): A Stable and Efficient Metal-Free Heterogeneous Photocatalyst

**DOI:** 10.1021/acsmacrolett.3c00217

**Published:** 2023-06-29

**Authors:** Sara Señorans, Isabel Valencia, Estíbaliz Merino, Marta Iglesias, Manuel A. Fernández-Rodríguez, Eva M. Maya

**Affiliations:** † Department of Frontiers in Materials Chemistry, Instituto de Ciencia de Materiales de Madrid (ICMM-CSIC), Sor Juana Inés de la Cruz, 3, Cantoblanco, Madrid 28049, Spain; § Universidad de Alcalá (IRYCIS), Departamento de Química Orgánica y Química Inorgánica, Instituto de Investigación Química “Andrés M. del Río” (IQAR), Campus Científico-Tecnológico, Facultad de Farmacia, Autovía A-II, Km 33.1, 28805-Alcalá de Henares, Madrid, Spain

## Abstract

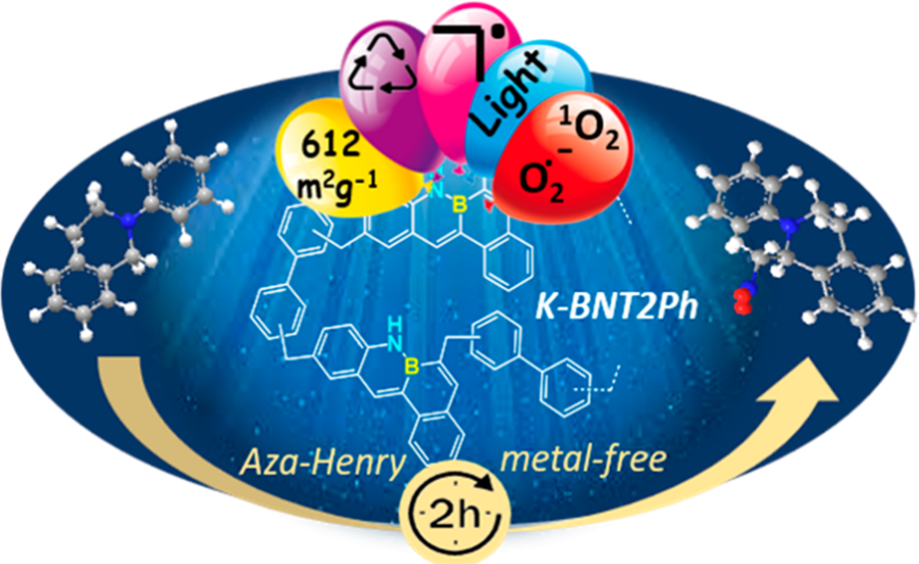

The first example of a porous polymer containing B–N covalent bonds, prepared from a tetraphene B–N monomer and biphenyl as a comonomer, is reported. It was prepared using the solvent knitting strategy, which allows the connection between the aromatic rings of the two monomers through methylene groups provided by an external cross-linking agent. The new polymer exhibited micromeso porosity with an *S*_BET_ of 612 m^2^/g, high thermal stability, and potential properties as a heterogeneous photocatalyst, since it is very active in the aza-Henry coupling reaction (>98% of conversion and selectivity). After the first run, the catalyst improves its photocatalytic activity, shortening the reaction time to only 2 h and maintaining this activity in successive runs. The presence of a radical in this structure that remains stable with successive runs makes it a new type of material with potential applications as a highly stable and efficient photocatalyst.

The incorporation of boron–nitrogen (B–N) covalent bonds, which are isoelectronic with the C=C bonds, in BN-polyaromatic hydrocarbons (BN-PAHs), also named azaborines, is now being intensively explored to modulate the electronic properties of these materials. The boron atom in B–N covalent bonds provides a coplanar conjugation due to its sp^2^ hybridization, which results in efficient charge transport.^1^ Thus, there are many synthetic procedures that have been developed to obtain the three possible isomers, 1,2-, 1,3-, or 1,4-azaborines (BN).^2,3^ Among the potential applications of these molecules, their use in biochemistry, pharmacology, catalysis, and optoelectronics is reported as the most promising.^2,3^ At the molecular level some of these applications have been investigated, for example as thermally activated delayed fluorescence-based OLEDs.^4^ However, only one example of the employment of a BN-arene as homogeneous photocatalyst has been described.^5^ The incorporation of B–N covalent bonds in polymers can generate interesting materials that combine the unique characteristics of BN-PAHs with the known properties of polymers, such as thermal and mechanical stability and especially processability. In this sense, only three types of polymers containing B–N covalent bonds have been reported (Figure 1a), but none of them is to be used as catalysts: BN-based polystyrene prepared from vinyl monomers containing BN units;^6−9^ BN-based conjugated polythiophenes with an application as organic field-effect transistors^10^ or in organic solar cells^11^ and BN-based polyphenylene-type polymers.^12^

**Figure 1:**
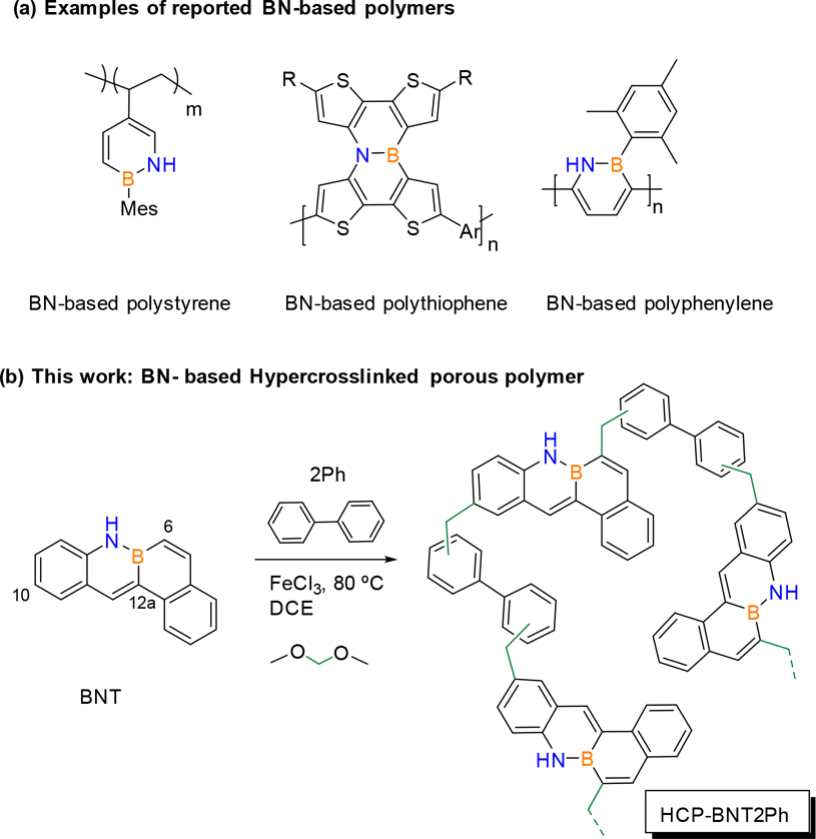
(a) BN-based polymers reported in the literature; (b) HCP BN-tetraphene polymer presented in this work.

To the best of our knowledge, no BN-PAH-based porous polymers have yet been reported. The development of porosity in polymers is a very interesting property because, in addition to providing mechanical and thermal stability to the structure, it can allow the lodging of molecules inside the pores, yielding interesting heterogeneous catalysts.^13^ Thus, porous organic polymers have positioned themselves as excellent photocatalysts because of combining the advantages of containing pores with those due to the cross-linked nature, including their versatility, stability, modifiability, and recyclability.^14−18^

In 2011, a new class of porous organic polymers that involves knitting rigid building blocks with an external cross-linker^19^ emerged with force on the scientific scenery due to its easy preparation method, simple but very versatile, allowing its properties modulation, and therefore, the possibility of many applications. They are prepared by C–H activations of aromatic monomers, without any previous functionalization, yielding hyper-cross-linked polymeric structures (HCPs). Thus, a large list of monomers has been employed to introduce different units in these polymers such as phenols, triptycene, triazine, fluoranthene, binaphthalene tetraphenylsilane, or tetraphenylgermanium, among others.^20^ However, no monomers containing B–N covalent bonds have been used to obtain hyper-cross-linked porous polymers. Thus, in this work we have polymerized the BN-tetraphene monomer (BNT, Figure 1b) with biphenyl (2Ph) as comonomer using the knitting solvent strategy to produce the first member of the BN-PAH-based hyper-cross-linked porous polymer: HCP-BNT2Ph.

In addition to the synthesis and full characterization of this polymer, its performance as a photocatalyst is reported, as a very promising application not explored until now with BN-PAH-based polymers.

HCP-BNT2Ph was prepared by mixing the BN-tetraphene monomer (BNT) (6a,7-dihydro-7-aza-6a-boratetraphene),^21^ with biphenyl in dry dichloroethane (DCE) as solvent and dimethoxymethane as a linker in the presence of FeCl_3_ as a catalyst. The details of the synthesis and purification are given in the Supporting Information (SI).

The incorporation of the BN-tetraphene unit in HCP-BNT2Ph was confirmed by elemental analysis (EA), nuclear magnetic resonance of boron and carbon (^11^B-NMR, ^13^C NMR), and infrared spectroscopy (FT-IR). The EA (Table S1, SI) indicated the presence of one BN-tetraphene unit for every two biphenyls, which points to greater reactivity of the biphenyl monomer with respect to the BN-tetraphene monomer as it was previously observed when biphenyl has polymerized with other monomers.^22,23^

The ^11^B-NMR solid-state spectrum (Figure 2a) confirms also the incorporation of the BN-tetraphene units, since it showed an intense signal around 0 ppm attributed to the B atoms of the network. The ^13^C NMR solid-state spectrum exhibited two groups of signals (Figure 2b). An intense one between 110 and 150 ppm is assigned to the aromatic CH and quaternary aromatic carbons, and a broad signal around 35 ppm is attributed to the methylene carbons that act as linkers.

**Figure 2:**
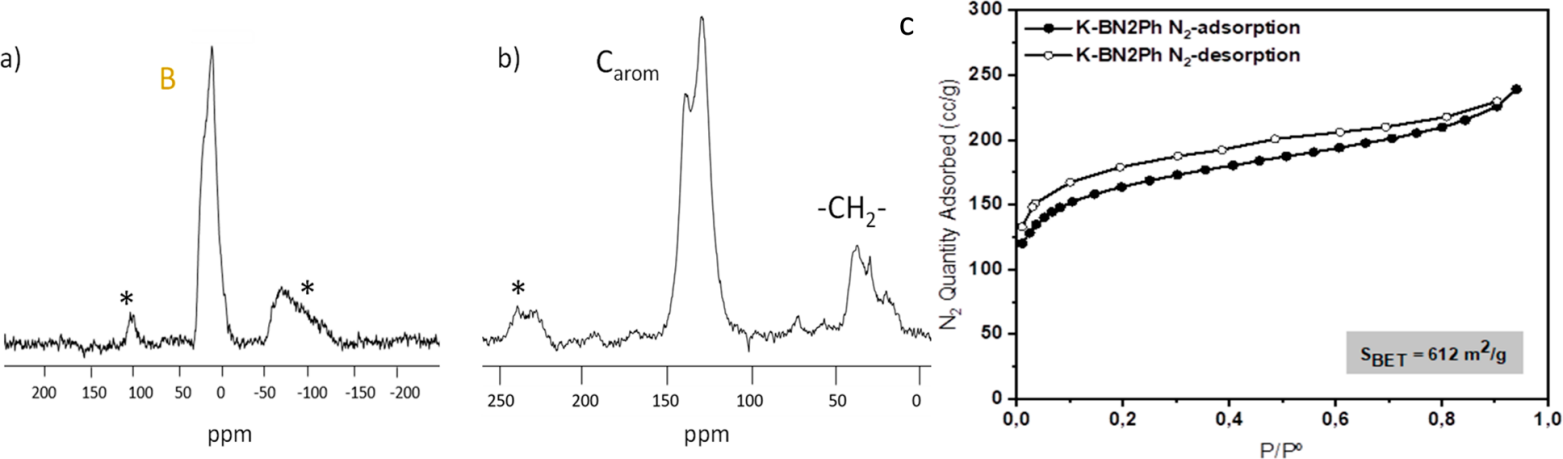
(a) ^11^B NMR; (b) ^13^C NMR solid state spectra (*) spinning bands; (c) N_2_ adsorption/desorption isotherms of HCP-BNT2Ph.

The FT-IR spectrum of HCP-BNT2Ph (Figure S2) showed four absorption peaks between 1215 and 917 cm^–1^. These bands could be attributed to stretching vibrations of the B–N bonds, observed also in the BNT monomer and detected between 1360 and 810 cm^–1^ in BN based compounds.^24^

We carried out a study by DFT calculations to shed light on the electron distribution in the BNT monomer (Figure S3). These natural charges were obtained by natural bond orbital analysis (NBO).^25^ In the BN derivative, all of the carbon atoms have a net negative charge except for C in the α position to the N atom with a value of the charge of +0.20987. According to the NBO method, the carbons 6, 10, and 12a in the BN-tetraphene monomer are the most activated positions for electrophilic aromatic substitutions; thus, the growth of the K-BNT2Ph network will probably occur through these positions. This fact, together with the polarization of the B–N bond, could generate an open structure.

The thermal stability was studied by TGA in N_2_ and O_2_ atmospheres (Figure S4). The new polymer showed high thermal stability, with degradation temperatures above 300 °C and two degradation steps. The first weight loss could be attributed to the break of the methylene groups that connect the aromatic rings, and the second one could be attributed to the generalized degradation of the polymer.

In an oxidizing atmosphere, an increase in weight is observed at about 300 °C that can be attributed to partial oxidation of the methylene groups that join the aromatic rings. This fact was previously observed in other HCP polymers^26,27^ and it agrees with the fact that the B–N tetraphene monomer yields an open structure with accessible methylene groups and therefore susceptible to oxidation.

The porous properties were evaluated by N_2_ adsorption–desorption isotherms (Figure 2c). The new material exhibited a specific surface area of 612 m^2^/g. As can be noticed, the amount of N_2_ adsorbed increased with the relative pressure as is characteristic of mesoporous materials; however, the isotherm shows a large N_2_ uptake at low pressure, which indicates the presence of micropores. In fact, the pore size distribution, calculated by the N_2_-DFT method (Figure S5), indicated the presence of pores around 2 nm. The morphology, observed by SEM (Figure S6) corresponds to the porous polymers with some agglomerates of spherical structures.^27,28^

The UV–vis spectrum in the suspension of the novel polymer indicates light absorption in the blue region of the spectrum, being the light that will be chosen for the evaluation of photocatalytic activity (420 nm; Figure S7).

The photocatalytic activity of the B–N polymer was evaluated through the aza-Henry cross-coupling reaction (the catalytic experiment’s details and catalytic activity monitoring are given in the SI). This model reaction is a very useful tool in organic syntheses to generate C–C bonds and is very extended to evaluate the photocatalytic activity of porous materials.^14,29^ The first essays were conducted using 2-phenyl-1,2,3,4-tetrahydroisoquinoline (THIQ) as the substrate and nitromethane as a nucleophile, without additional solvent and blue LED as the light source under an air atmosphere (Table 1). After carrying out several experiments varying the amount of catalyst and the reaction time, a catalyst loading of 5 mol % and blue light irradiation for 4 h (entry 1) was established as optimal reaction conditions to carry out this transformation. Under these conditions, complete conversion of the substrate and high selectivity toward the coupling product was achieved. Notably, the ketone (**B** in Table 1), which is often formed in this reaction due to the oxidizing atmosphere, was not detected in any of the tests performed.

**Table 1:**
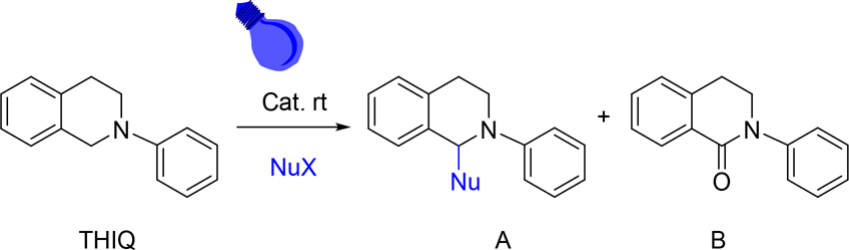
C–C aza-Henry Coupling Reaction of THIQ^a^

	NuX	oxidant	*t* (h)	conv.^e^ (%)	Sel. A^e^ (%)
1	HCH_2_NO_2_	air	4	>98	>98
2^b^	HCH_2_NO_2_	air	2	>98	>98
3^c^	HCH_2_NO_2_	air	4	17	>98
4	HCH_2_NO_2_	N_2_	4	14	>98
5	HCH_2_NO_2_	air, dark	4	0	0
6^d^	HCH_2_NO_2_	air	4	18	>98
7	HCH(COCH_3_)_2_	air	4	33	>98
8^b^	HCH(COCH_3_)_2_	air	2	>98	>98
9	TMSCN	air	4	40.5	>98
10^b^	TMSCN	air	2	>98	>98

a Reaction conditions: THIQ (314 mg, 1.5 mmol), nucleophile (115 mmol), catalyst (40 mg, 5 mmol %, based on the repeat unit), blue LED (50 W).

b Second run.

c Without catalyst.

d Monomer BNT as catalyst.

e Average of three experiments.

The recycled catalyst (entry 2) yielded the target compound in only 2 h. This fact indicates that the polymer can be modified upon irradiation for certain hours, improving its catalytic performance. The kinetic profiles of both runs were recorded (Figure 3) confirming a higher reaction rate in the second run.

**Figure 3:**
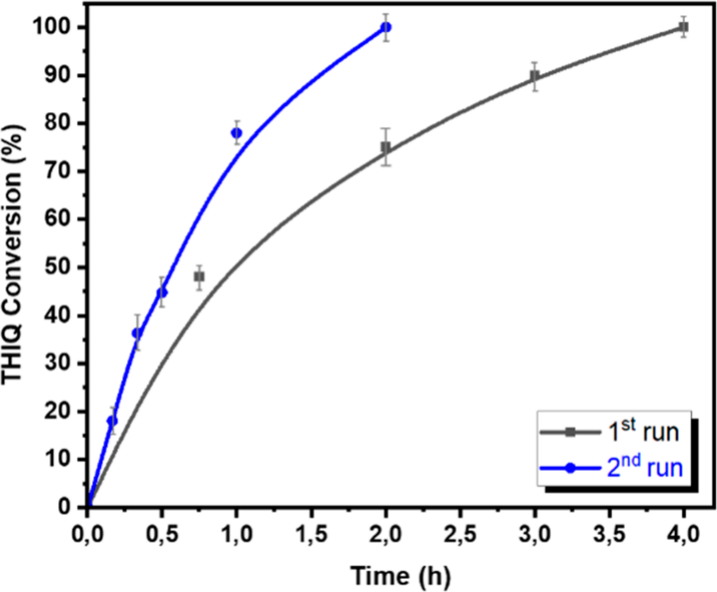
Kinetic profiles of HCP-BNT2Ph in the first and second run in the aza-Henry coupling of THIQ and nitromethane.

Control experiments without catalyst (entry 3; conversion of 17%), in the absence of oxygen (entry 4, conversion of 14%), and in the dark (entry 5), showed low or no evolution to the aza-Henry product. These results indicate that all components are needed to promote electron transfer and thus the reaction. Additionally, the monomer BNT was also used as a catalyst (entry 6). It was observed that a very low THIQ conversion for the soluble catalyst, which was attributed to very low adsorption at the irradiation wavelength (Figure S8). From the Kubelka–Munk function equation (tauc plot) obtained by the UV–vis diffuse reflectance spectra (Figure S9), a band gap energy of 2.38 eV was obtained, which is a value similar to those reported for conjugated porous polymers^14^ and within the interval obtained for efficient photocatalysts recently reported.^29^

To gain more insight into the applicability of the BNT-polymer as a photocatalyst, other nucleophiles, such as malonate (entries 7 and 8) and trimethylsilyl cyanide (entries 9 and 10), were tested. In both cases, the target compound was obtained with almost 100% selectivity and full THIQ conversion in the second run. Figures S10–S12 showed the crude ^1^H NMR spectra of product A obtained in reactions 1, 8, and 10. As can be noticed, all spectra are very clean, with no traces of THIQ (Figure S13) or amide subproduct **B** in any case.

To verify the heterogeneous character of the new catalyst, recyclability studies were carried out using two different substrates, nitromethane (Figure 4) and trimethylsilyl cyanide (Figure S14). When nitromethane was used as a nucleophile, after the first run with 4 h of irradiation, the catalyst was filtered off on a Büchner funnel, washed with acetone, and dried at 90–100 °C under vacuum overnight. The next reactions were run for 2 h. As can be observed, the catalyst maintained its activity for at least 5 consecutive runs. In the case of trimethylsilyl cyanide, the reactions after the third run needed more time, probably due to the adsorption of reactants within the pores.

**Figure 4:**
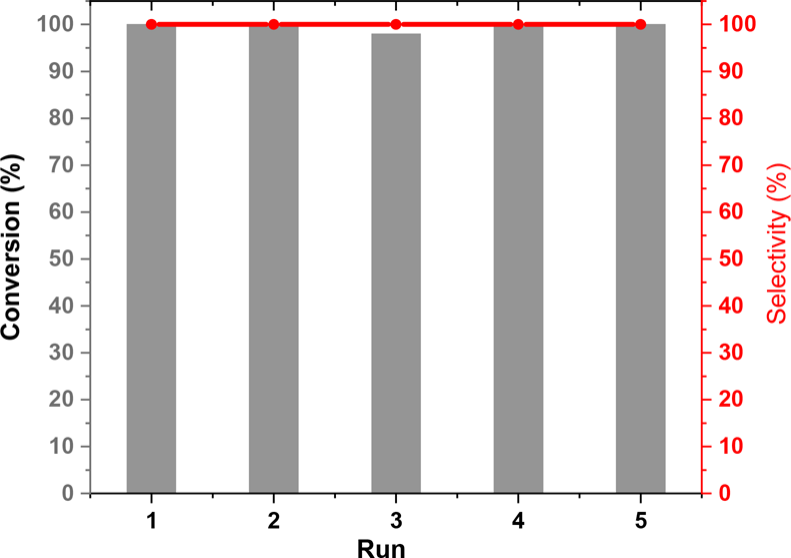
Recyclability of HCP-BNT2Ph in the aza-Henry reaction between THIQ and nitromethane: Run 1, 4 h of irradiation; Runs 2–5, 2 h of irradiation.

The polymer did not undergo any significant change in its structure as could be verified by elemental analysis, infrared spectroscopy analysis, SEM, and UV–vis recorded after the last recycling (Table S1 and Figures S2, S6, and S7, respectively). The N_2_ adsorption/desorption isotherm has a similar shape to the fresh catalyst but a decrease in the specific surface BET area was observed (Figure S15) as it was previously reported in porous organic polymers after certain reactions.^20^ In our case, no decrease in the catalytic activity is observed, which could be indicating that the active centers are not altered after the catalytic activity and are probably located more on the outer surfaces than inside the pores. UV–vis spectrum recorded after recycling did not show significant change either (Figure S7).

To understand the photocatalytic behavior between the runs, electron paramagnetic resonance (EPR) spectra of the starting and recovered catalyst were recorded (Figure 5). Both spectra showed the appearance of a single-line Lorentzian signal, characteristic of unpaired electrons in conjugated structures. The presence of the same signal in the recycled catalyst indicated the stability of the radical anion. In addition to the stable radical of the catalyst, the oxygen species formed during the oxidation process were investigated. Thus, different scavengers were added to the reaction mixture (CuSO_4_ (e^–^), *p*-benzoquinone (O_2_), and DABCO (^1^O_2_) (Figure S16). CuSO_4_ has no significant impact on the reaction, *p*-benzoquinone partially reduces the conversion, and in the presence of DABCO a loss in the selectivity was observed. These results indicate that both radical (O_2_˙) and singlet oxygen (^1^O_2_) species are involved in the reaction.^30,31^ Finally, the HCP-BNT2Ph photocatalyst was compared with other reported metal-free porous organic polymers used in the same model reaction, where 2-phenyl-1,2,3,4-tetrahydroisoquinoline (THIQ) was used as the substrate and nitromethane as the nucleophile and solvent (Table S2).

**Figure 5:**
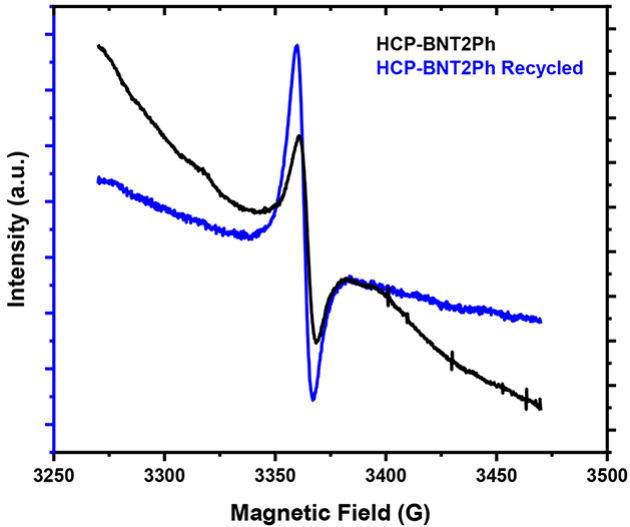
Electronic paramagnetic resonances (EPR) of HCP-BNT2Ph and HCP-BNT2Ph after 5 runs.

As it can be noticed, although the irradiation conditions are not comparable, HCP-BNT2Ph is the fastest photocatalyst since it produces the coupling derivative in a much shorter time (2–4 h) than those published so far (10–48 h), also achieving complete conversion of THIQ.

In summary, a metal-free heterogeneous photocatalyst based on a porous polymer containing BN covalent bonds has been reported to be more efficient than other porous polymers reported for the aza-Henry coupling. In addition, the synthetic strategy used, the knitting solvent approach, opens the possibility of expanding this family by using other BN-monomers that can be combined with other photoactive units, enhancing their properties as photocatalysts.

## Supporting Information Available

Materials and methods; Natural population analysis; Cartesian coordinates; DFT calculations; Figures S1–S16; Tables S1 and S2; Supporting references (PDF)

## References

[ref1] Liu X.; Pang S.; Zeng L.; Deng W.; Yang M.; Yuan X.; Li J.; Duan C.; Huang F.; Cao Y. An electron acceptor featuring a B-N covalent bond and small singlet-triplet gap for organic solar cells. Chem. Commun. 2022, 58 (62), 8686–8689. 10.1039/D2CC03172H. 35833246

[ref2] Abengózar A.; García-García P.; Fernández-Rodríguez M. A.; Sucunza D.; Vaquero J. J. Recent developments in the chemistry of BN-aromatic hydrocarbons. Adv. Heterocycl. Chem. 2021, 135, 197–259. 10.1016/bs.aihch.2021.01.001.

[ref3] Giustra Z. X.; Liu S. Y. The State of the Art in Azaborine Chemistry: New Synthetic Methods and Applications. J. Am. Chem. Soc. 2018, 140 (4), 1184–1194. 10.1021/jacs.7b09446. 29314835 PMC6190836

[ref4] Suresh S. M.; Duda E.; Hall D.; Yao Z.; Bagnich S.; Slawin A. M. Z.; Bassler H.; Beljonne D.; Buck M.; Olivier Y.; Kohler A.; Zysman-Colman E. A Deep Blue B,N-Doped Heptacene Emitter That Shows Both Thermally Activated Delayed Fluorescence and Delayed Fluorescence by Triplet-Triplet Annihilation. J. Am. Chem. Soc. 2020, 142, 6588–6599. 10.1021/jacs.9b13704. 32134259

[ref5] Kondo M.; Agou T. Catalytic aerobic photooxidation of triarylphosphines using dibenzo-fused 1,4-azaborines. Chem. Commun. 2022, 58, 5001–5004. 10.1039/D2CC00782G. 35362494

[ref6] Van de Wouw H. L.; Lee J. Y.; Klausen R. S. Gram-scale free radical polymerization of an azaborine vinyl monomer. Chem. Commun. 2017, 53 (53), 7262–7265. 10.1039/C7CC02300F. 28590472

[ref7] Thiedemann B.; Gliese P. J.; Hoffmann J.; Lawrence P. G.; Sönnichsen F. D.; Staubitz A. High molecular weight poly (N-methyl-B-vinylazaborine) - A semi-inorganic B-N polystyrene analogue. Chem. Commun. 2017, 53 (53), 7258–7261. 10.1039/C6CC08599G. 28054054

[ref8] Wan W. M.; Baggett A. W.; Cheng F.; Lin H.; Liu S. Y.; Jäkle F. Synthesis by free radical polymerization and properties of BN-polystyrene and BN-poly (vinylbiphenyl). Chem. Commun. 2016, 52 (93), 13616–13619. 10.1039/C6CC07332H. 27812559

[ref9] Lin H.; McConnell C. R.; Jilus B.; Liu S.-Y.; Jakle F. Changing up BN-Polystyrene: Effect of Substitution Pattern on the Free-Radical Polymerization and Polymer Properties. Macromolecules 2019, 52 (12), 4500–4509. 10.1021/acs.macromol.9b00466.

[ref10] Wang X. Y.; Zhuang F. D.; Wang J. Y.; Pei J. Incorporation of polycyclic azaborine compounds into polythiophene-type conjugated polymers for organic field-effect transistors. Chem. Commun. 2015, 51 (99), 17532–17535. 10.1039/C5CC06927K. 26452002

[ref11] Pang S.; Wang Z.; Yuan X.; Pan L.; Deng W.; Tang H.; Wu H.; Chen S.; Duan C.; Huang F.; Cao Y. A Facile Synthesized Polymer Featuring B-N Covalent Bond and Small Singlet-Triplet Gap for High-Performance Organic Solar Cells. Angew. Chem.- Int. Ed. 2021, 60 (16), 8813–8817. 10.1002/anie.202016265. 33682269

[ref12] Ahmadi Y.; Kim K.-H. Recent Progress in the Development of Hyper Cross-Linked Polymers for Adsorption of Gaseous Volatile Organic Compounds. Polym. Rev. 2023, 63 (2), 365–393. 10.1080/15583724.2022.2082470.

[ref13] Zhang Y.; Riduan S. N. Functional porous organic polymers for heterogeneous catalysis. Chem. Soc. Rev. 2012, 41 (6), 2083–2094. 10.1039/C1CS15227K. 22134621

[ref14] Zhang T.; Xing G.; Chen W.; Chen L. Porous organic polymers: A promising platform for efficient photocatalysis. Mater. Chem. Front. 2020, 4 (2), 332–353. 10.1039/C9QM00633H.

[ref15] Debruyne M.; Van Speybroeck V.; Van Der Voort P.; Stevens C. V. Porous organic polymers as metal free heterogeneous organocatalysts. Green Chem. 2021, 23, 7361–7434. 10.1039/D1GC02319E.

[ref16] Chakraborty J.; Nath I.; Song S.; Mohamed S.; Khan A.; Heynderickx P. M.; Verpoort F. Porous organic polymer composites as surging catalysts for visible-light-driven chemical transformations and pollutant degradation. J. Photochem. Photobiol. C 2019, 41, 100319 10.1016/j.jphotochemrev.2019.100319.

[ref17] Kumar G.; Cai B.; Ott S.; Tian H. Visible-light photoredox catalysis with organic polymers. Chem. Phys. Rev. 2023, 4, 011307 10.1063/5.0123282.

[ref18] Zhang Z.; Jia J.; Zhi Y.; Ma S.; Liu X. Porous organic polymers for light-driven organic transformations. Chem. Society Rev. 2022, 51, 2444–2490. 10.1039/D1CS00808K. 35133352

[ref19] Li B.; Gong R.; Wang W.; Huang X.; Zhang W.; Li H.; Hu C.; Tan B. A new strategy to microporous polymers: Knitting rigid aromatic building blocks by external cross-linker. Macromolecules 2011, 44 (8), 2410–2414. 10.1021/ma200630s.

[ref20] Valverde-González A.; Iglesias M.; Maya E. M. Metal Catalysis with Knitting Aryl Polymers: Design, Catalytic Applications, and Future Trends. Chem. Mater. 2021, 33 (17), 6616–6639. 10.1021/acs.chemmater.1c01569.

[ref21] Valencia I.; García-García P.; Sucunza D.; Mendicuti F.; Vaquero J. J. 1,10a-Dihydro-1-aza-10a-boraphenanthrene and 6a,7-Dihydro-7-aza-6a-boratetraphene: Two New Fluorescent BN-PAHs. J. Org. Chem. 2021, 86 (23), 16259–16267. 10.1021/acs.joc.1c01095. 34806882 PMC8650019

[ref22] Valverde-González A.; Guan L. Z.; Ferrer M. L.; Iglesias M.; Maya E. M. Iron Phthalocyanine-Knitted Polymers as Electrocatalysts for the Oxygen Reduction Reaction. ACS Appl. Mater. Interfaces 2020, 12 (29), 32681–32688. 10.1021/acsami.0c07412. 32578975

[ref23] Maya E. M.; Valverde-González A.; Iglesias M. Conversion of CO_2_ into Chloropropene Carbonate Catalyzed by Iron (II) Phthalocyanine Hypercrosslinked Porous Organic Polymer. Molecules 2020, 25, 4598 10.3390/molecules25204598. 33050266 PMC7587207

[ref24] Kong D.; Zhang D.; Guo H.; Zhao J.; Wang Z.; Hu H.; Xu J.; Fu C. Functionalized boron nitride nanosheets/poly (L-lactide) nanocomposites and their crystallization behavior. Polymers 2019, 11 (3), 440 10.3390/polym11030440. 30960424 PMC6473543

[ref25] For further information, see the Supporting Information.

[ref26] Sanz-Pérez E. S.; Rodríguez-Jardón L.; Arencibia A.; Sanz R.; Iglesias M.; Maya E. M. Bromine pre-functionalized porous polyphenylenes: New platforms for one-step grafting and applications in reversible CO_2_ capture. J. CO_2_ Util. 2019, 30, 183–192. 10.1016/j.jcou.2019.02.005.

[ref27] Guadalupe J.; Ray A. M.; Maya E. M.; Gómez-Lor B.; Iglesias M. Truxene-based porous polymers: From synthesis to catalytic activity. Polym. Chem. 2018, 9 (36), 4585–4595. 10.1039/C8PY01082J.

[ref28] Verde-Sesto E.; Maya E. M.; Lozano Á. E.; de la Campa J. G.; Sánchez F.; Iglesias M. Novel efficient catalysts based on imine-linked mesoporous polymers for hydrogenation and cyclopropanation reactions. J. Mater. Chem. 2012, 22 (47), 24637–24643. 10.1039/c2jm34927b.

[ref29] Fuerte-Díez B.; Valverde-González A.; Pintado-Sierra M.; Díaz U.; Sánchez F.; Maya E. M.; Iglesias M. Phenyl Extended Naphthalene-Based Covalent Triazine Frameworks as Versatile Metal-Free Heterogeneous Photocatalysts. Solar RRL 2022, 6 (2), 2100848 10.1002/solr.202100848.

[ref30] Bartling H.; Eisenhofer A.; Konig B.; Gschwind R. M. The Photocatalyzed Aza-Henry Reaction of N-Aryltetrahydroisoquinolines: Comprehensive Mechanism, H^•^- versus H^+^-Abstraction, and Background Reactions. J. Am. Chem. Soc. 2016, 138 (36), 11860–11871. 10.1021/jacs.6b06658. 27541322

[ref31] Liu Q.; Li Y. N.; Zhang H. H.; Chen B.; Tung C. H.; Wu L. Z. Reactivity and mechanistic insight into visible-light-induced aerobic cross-dehydrogenative coupling reaction by organophotocatalysts. Chem.—Eur. J. 2012, 18 (2), 620–627. 10.1002/chem.201102299. 22162148

