# Supplementary material for: Hyper-Cross-Linked Porous Polymer Featuring B–N Covalent Bonds (HCP-BNs): A Stable and Efficient Metal-Free Heterogeneous Photocatalyst
Source: ACS Macro Lett. 2023 Jun 29;12(7):949–54. doi: 10.1021/acsmacrolett.3c00217 (PMC10357577; doi:10.1021/acsmacrolett.3c00217)
Supplement: Supplementary file 1 — mz3c00217_si_001.pdf [file mz3c00217_si_001.pdf]

Supporting Information

Hypercrosslinked porous polymer featuring B-N covalent bonds (HCP-BNs): a stable and efficient metal-free heterogeneous photocatalyst

Sara Señorans,[a] Isabel Valencia,[b] Estíbaliz Merino,[b] Marta Iglesias,[a] Manuel A. Fernández-Rodríguez,[b*] Eva M. Maya[a*]

[a]Instituto de Ciencia de Materiales de Madrid (ICMM-CSIC), Departamento de Nuevas Arquitecturas en Quimica de Materiales, Sor Juana Inés de la Cruz, 3, Cantoblanco. Madrid 28049, Spain, email: eva.maya@csic.es
[b]Universidad de Alcalá (IRYCIS). Departamento de Química Orgánica y Química Inorgánica, Instituto de Investigación Química "Andrés M. del Río" (IQAR). Campus Científico-Tecnológico, Facultad de Farmacia, Autovía A-II, Km 33.1, 28805-Alcalá de Henares, Madrid (Spain), e-mail: mangel.fernandezr@uah.es

Materials and methods

6a,7-Dihydro-7-aza-6a-boratetraphene, BN-Tetraphene monomer (BNT) was prepared according with the method previously reported (1).

1,2- Benzanthracene, tetraphene monomer (T) was supplied by Acros Organics (99%).

2-Phenyl-1,2,3,4-tetrahydroisoquinoline was synthetized from 1,2,3,4-tetrahydroisoquinoline following the procedure described (2).

Solvents were dried by elution through a PureSolv Innovative Technology column drying system.

ATR-FTIR spectra were recorded (cm^{-1}) on a PerkinElmer Spectrum Two spectrometer with a Fourier equipped with a diamond internal element.

^{13}C solid-state MAS-NMR measurements were recorded with a Bruker AV400 WB spectrometer.

Thermogravimetric and differential thermal analyses (TGA-DTA) were conducted on a with a TA Instruments Model TA-Q500 analyzer. The samples were heated from 40 to 800 ºC under N_2 atmosphere with a heating rate 10 ºC/min.

Nitrogen adsorption isotherms where measured at 77 K using a Micromeritics ASAP 2020 M surface and porosity analyzer. Prior to measurement, the samples were degassed for 12 h at 100 °C.

The morphology was analyzed by a Field Emission Scanning Electron Microscopy SEM model Hitachi S-8000 equipped with an Energy Dispersive X-ray spectroscopy (EDAX) analyzer, EDAX SUTW equipment (Super Ultra-Thin Window).

Ultraviolet–visible diffuse reflectance spectra (UV– Vis DRS) were carried out on a Shimadzu UV-2401 PC UV-Visible Spectrometer. The reflectance values (R) were transformed to the Kubelka–Munk function F(R) according to the eq (1): The band gap was estimated by a Tauc plot of (hνF(R))1/n against hν (h is Planck's constant, ν is the frequency of vibration and n = 1/2 for direct transitions).

The photoreactor used the reactions tested was lab-made and has one LED blue lamps of 50 W. The progress of the C-C aza Henry reactions was followed by ^{1}H-NMR in Varian Inova 300 MHz and a JEOL JNM-ECZ400R.

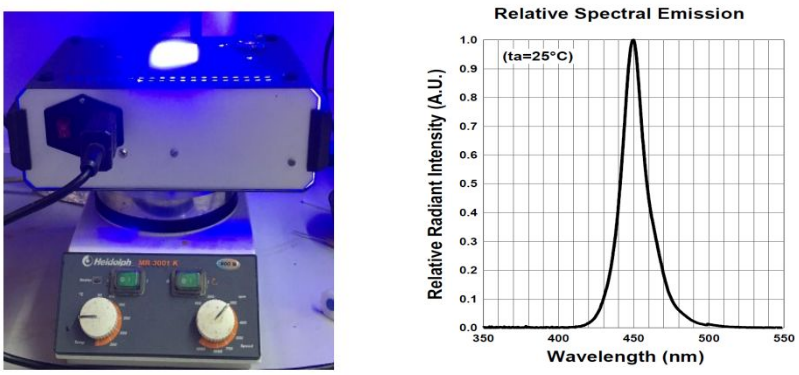

Figure S1. Photoreactor used (lab-made) and Light-emitting spectrum of the used blue LEDs.

Synthesis of photocatalyst HCP-BNT2Ph

A solution of 0.5 mmol of BN-tetraphene monomer (BNT) and 0.5 mmol of biphenyl in 10 mL of anhydrous dichloroethane was placed in a Schlenk. The solution was purged with nitrogen over 20 min and then 4 mmol of dimethoxymethane and 2 mmol of $FeCl_3$ were carefully added. The mixture was stirred for 48 h at 80°C. The resulting dark brown precipitate was collected by filtration and washed with ammonia under stirring for 12 h

to remove unreacted $FeCl_3$. Then, the suspension was washed under stirring with HCl for 12h and with water 4 h. The dark brown solid was washed with hot methanol for 12 h. Finally, the polymer was washed with THF under stirring. A green product was obtained as fine powder and was dried at 100°C overnight.

DFT Calculations

The reported structures were optimized at Density Functional Theory level as implemented in Gaussian 16 (3). The geometry optimizations were performed using M062X functional (4) with 6-311++g(d,p) basis set for all atoms. Solvent effects were considered in the calculations applying the polarizable continuum model (IEFPCM) using 1,2-dichloroethane as solvent at 273.15 K. The bonding situation in the complexes was studied using Natural Bond Orbital (5) analysis (NBO, 3.1 version) (6). Main atomic charges (NPA) (7) have been calculated.

Natural Population Analysis

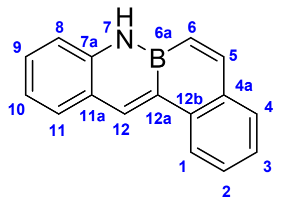

C1	-0.19153
C2	-0.21824
C3	-0.22396
C4	-0.19818
C4a	-0.07306
C5	-0.14937
C6	**-0.50037**
B6a	0.71649
N7	**-0.75436**
C7a	0.20987
C8	-0.24997
C9	-0.19262
C10	**-0.25178**
C11	-0.17397
C11a	-0.13502
C12	-0.08808
C12a	**-0.34365**
C12b	-0.05036

CARTESIAN COORDINATES OF THE COMPUTED STRUCTURE

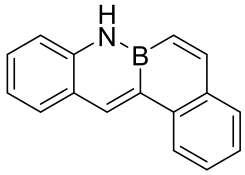

C	-5.93060900	-1.55527900	-0.24155700
C	-4.58422800	-1.64782200	-0.24269600
C	-4.54479300	0.99802300	-0.24782000
C	-6.01540100	0.96725300	-0.25504500
C	-6.66945100	-0.29089600	-0.24867500
C	-3.80521800	2.14792800	-0.23818200
C	-2.36962400	2.13887200	-0.23561100
C	-1.65854900	0.91391800	-0.23968100
C	-0.25000900	0.92272600	-0.23927500
H	0.28035800	-0.02404200	-0.24279100
C	0.43574900	2.11787900	-0.23469200
C	-0.25719900	3.34295900	-0.22989500
C	-1.63392700	3.34502100	-0.23019100
H	-1.79636100	-1.10805000	-0.24199700
H	-6.55158100	-2.44953000	-0.23719500
H	-4.13519100	-2.63865200	-0.23920100
H	-4.26585200	3.13365400	-0.23085800
H	1.51985900	2.11222500	-0.23471800
H	0.29244500	4.27641600	-0.22619800
H	-2.18320000	4.28103000	-0.22683100
C	-6.80810700	2.12709300	-0.27065800
H	-6.33579000	3.10226100	-0.28253100
C	-8.19177800	2.05862700	-0.27451300
H	-8.77574300	2.97198300	-0.28640200
C	-8.83326900	0.81697700	-0.26396800
C	-8.07322800	-0.33956700	-0.25224900
H	-8.55952600	-1.30992300	-0.24634300
H	-9.91561400	0.75988400	-0.26650800
B	-3.78274000	-0.33643800	-0.24588700
N	-2.36177800	-0.26572700	-0.24297500

Catalytic activity

General procedure:

The aza-Henry couplings were done in a glass microreactor. 2-phenyl-1,2,3,4-tetrahydroisoquinoline (314 mg, 1.5 mmol), K-BNT2Ph (40 mg, 5 mmol %) and the corresponding nucleophile (115 mmol) were added. The reaction mixture was purged with air and maintained with an air or N_2 balloon (see Table 2). The mixture was stirred

under blue LED light (50 W) irradiation, the time indicated in Table 1 (4h or 2h). The catalyst was separated by filtration and the solvent was removed by rotation under vacuum. The residue was analyzed by ^{1}H-NMR.

Recycling experiments:

The recyclability of K-BNT2Ph was evaluated using nitromethane as a nucleophile. After completion of the first reaction (4 h), the K-BNT2Ph catalyst was separated from the reaction medium by Büchner funnel, washed with acetone, and dried at 90-100 °C under vacuum overnight. Afterward, it was used in a new reaction for 2 hours, since when studying its kinetics, it is observed that it reaches the maximum conversion in that time. The solvent was removed after each operation as above and the corresponding residues were analyzed by ^{1}HNMR. This procedure was repeated three more times.

Figures

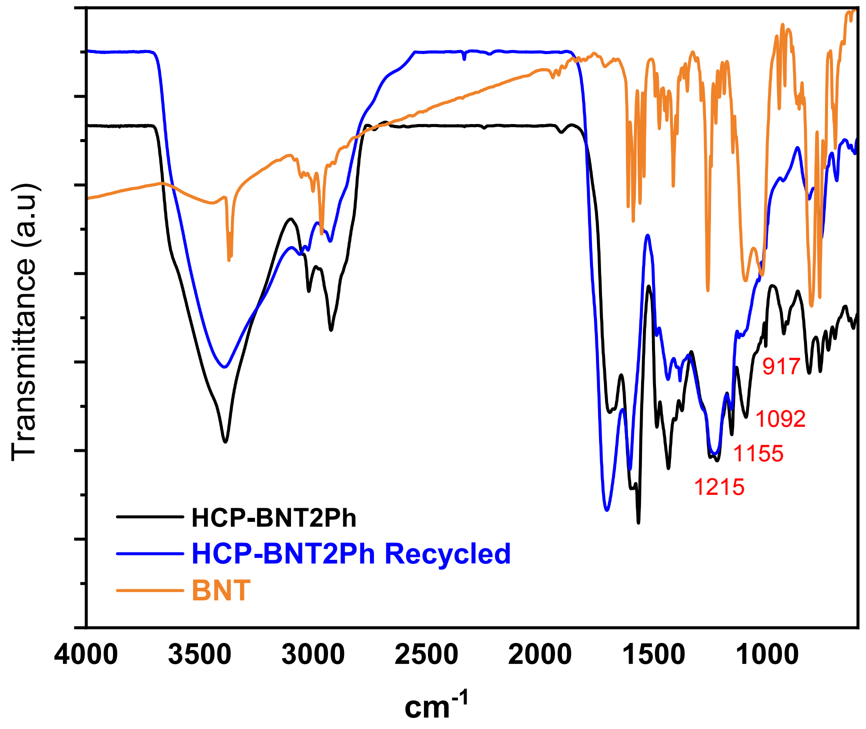

Figure S2. FT-IR spectra of HCP-BNT2Ph and after being reused in five runs and monomer BNT

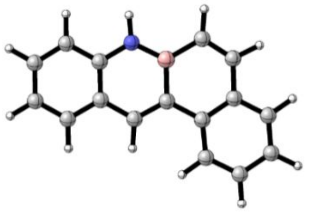

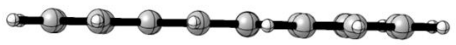

Figure S3. Geometry optimization of BNT monomer by DFT calculations (M062X/6-311++g(d,p)).

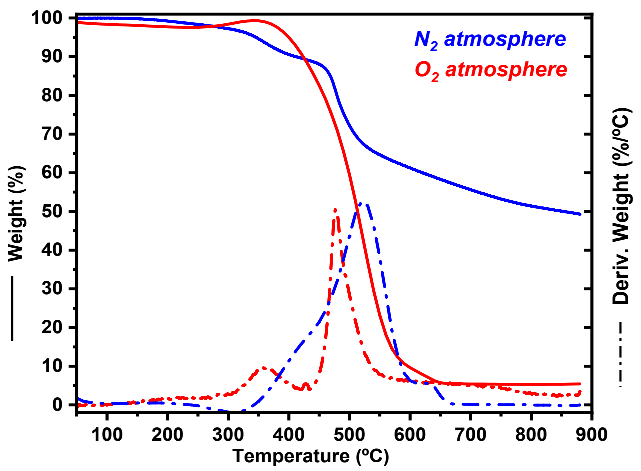

Figure S4. TGA (dot lines) and DGTA (dash-dot lines) of HCP-BNT2Ph in N_2 and O_2 atmosphere.

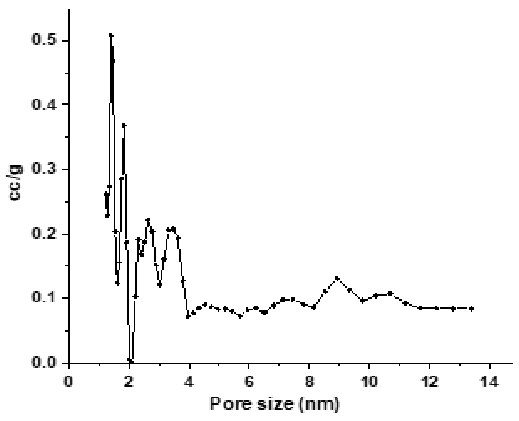

Figure S5. Pore size distribution of HCP-BNT2Ph

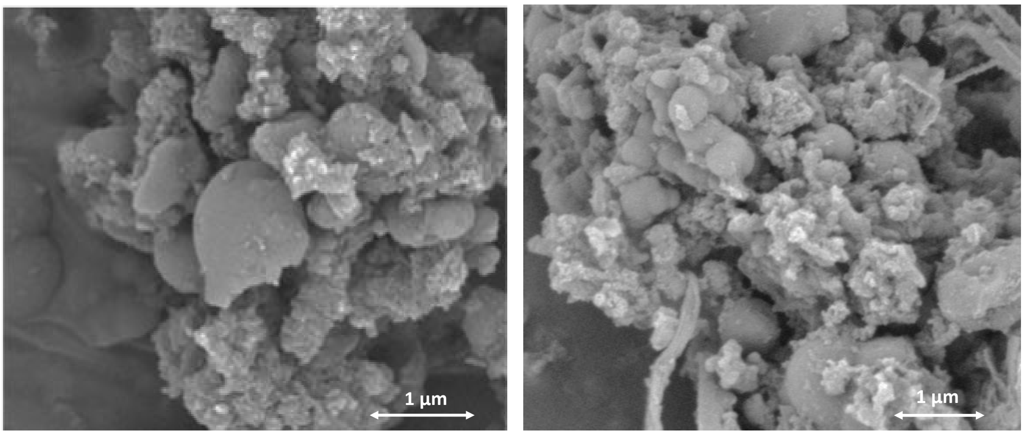

Figure S6. SEM image of HCP-BNT2Ph (left) and after being reused in five runs (right)

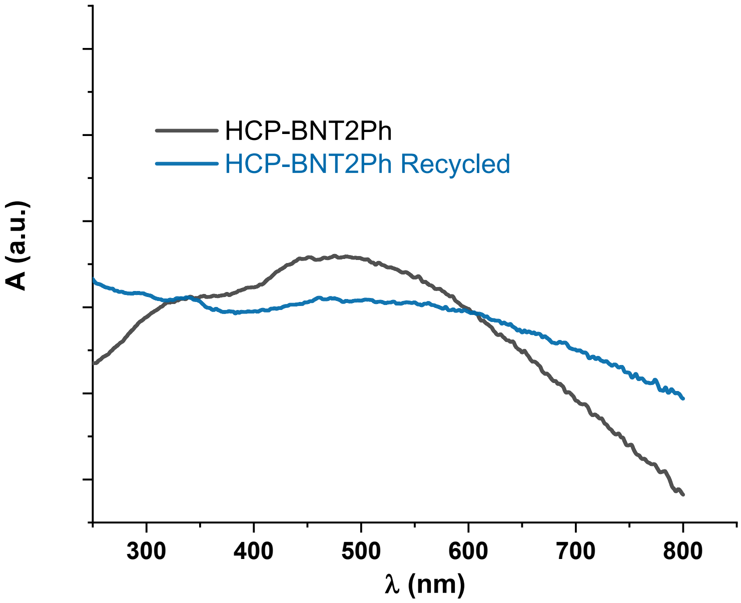

Figure S7. UV-Vis spectra (suspension) of HCP-BNT2Ph and after being reused in five consecutive runs

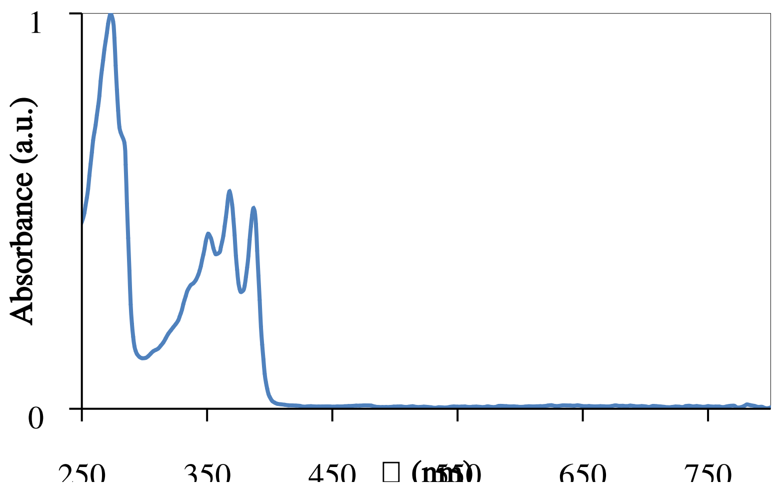

Figure S8. Absorption spectrum of the monomer BNT.

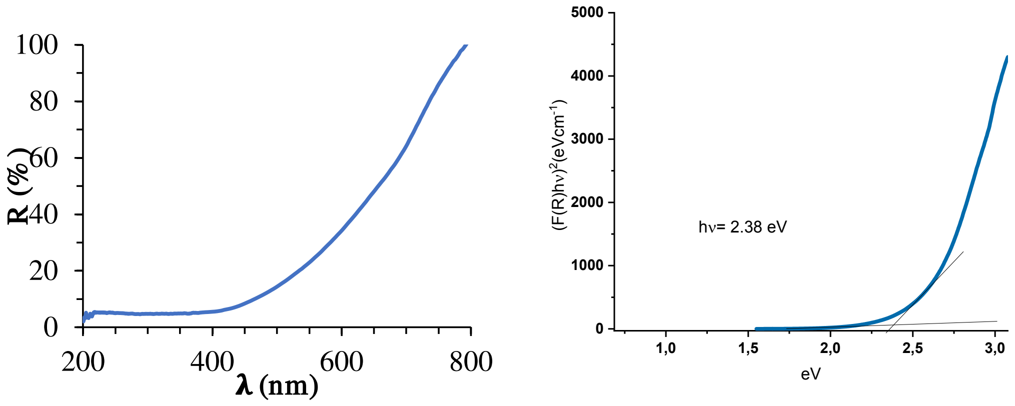

Figure S9. UV-Vis diffuse reflectance spectra (lefts) of HCP-BNT2Ph and tauc-plot (right).

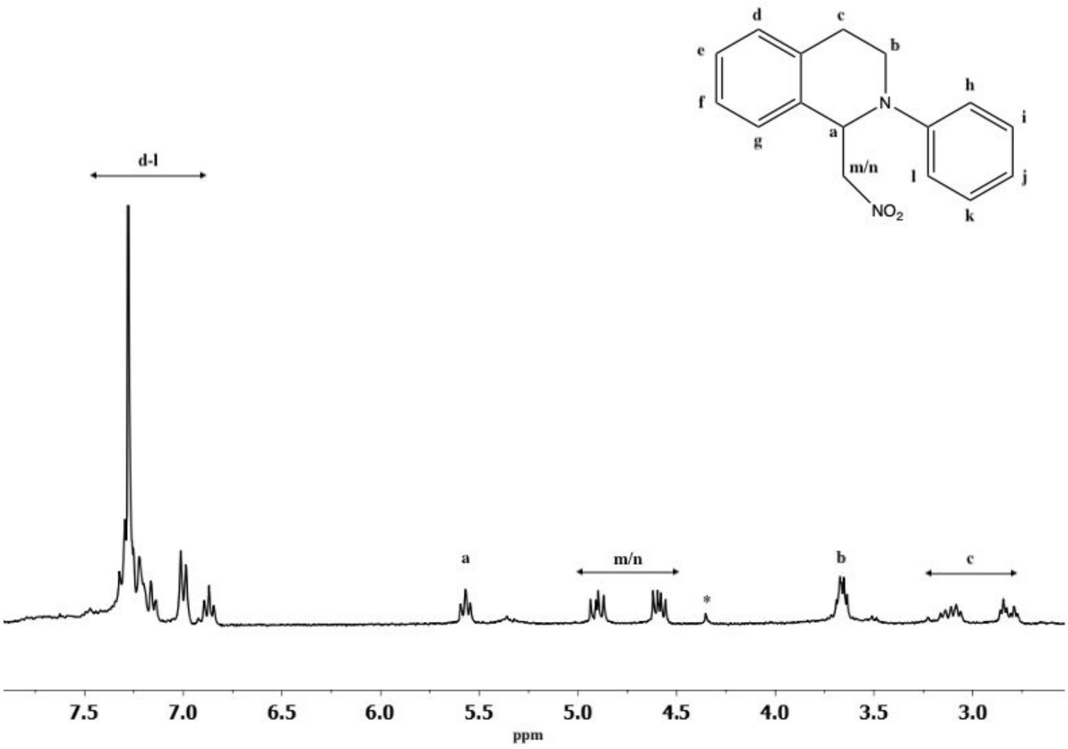

Figure S10. ^{1}H-NMR of 1-(nitromethyl)-2-phenyl-1,2,3,4-tetrahydroisoquinoline (Product A, Table 2, entry 1)

^{1}H-NMR (300 MHz, $CDCl_3$) δ 6.85–7.45 (m, 9H $H_{d\text{-}l}$), 5.54 (dd, 1H, H_a), 4.92–4.51 (m, 2H, $H_{m/n}$), 3.58 (m, 2H, H_b), 3.20–2.75 (m, 2H, H_c). These data is fully consistent with the data reported in the literature for 1-(nitromethyl)-2-phenyl-1,2,3,4-tetrahydroisoquinoline (8).

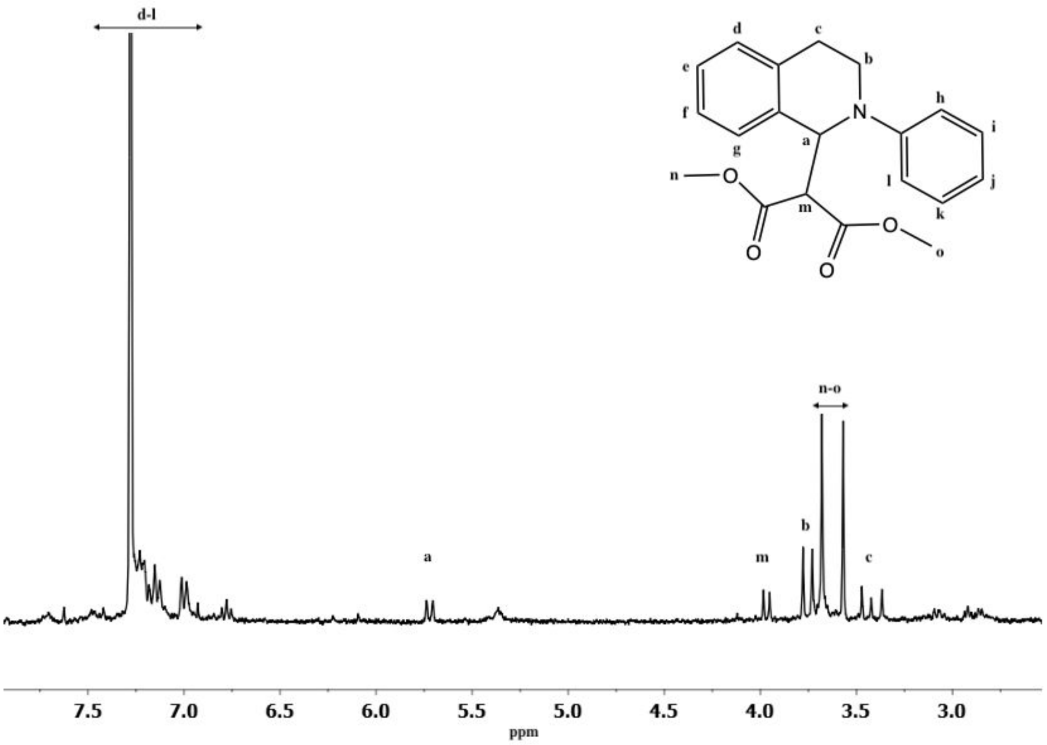

Figure S11. ^{1}H-NMR of dimethyl 2-(2-phenyl-1,2,3,4-tetrahydroisoquinolin-1yl)malonate (Product A, Table 2, entry 7)

^{1}H-NMR (300 MHz, CDCl3) δ 7.30–6.75 (m, 9H, $H_{d\text{-}l}$), 5.75 (d, 1H, H_a), 3,90 (d, 1H, H_m), 3.75 (d, 2H, Hb), 3.70 (s, 3H, $H_{n/o}$), 3.55 (s, 3H, $H_{n/o}$), 3.45 (d, 2H, H_c). These data is fully consistent with the data reported in the literature for dimethyl 2-(2-phenyl-1,2,3,4-tetrahydroisoquinolin-1yl)malonate (8).

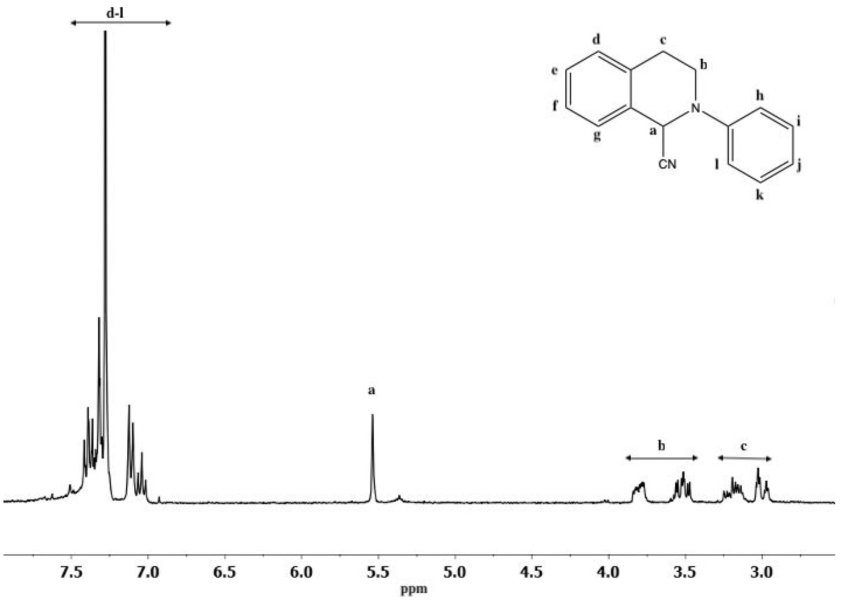

Figure S12. ^{1}H-NMR of 2-phenyl-1,2,3,4-tetrahydroisoquinolin-1-carbonitrile (Product A, Table 2, entry 9)

^{1}H-NMR (300 MHz, $CDCl_3$) δ 7.40–7.02 (m, 9H, $H_{d\text{-}l}$), 5.52 (s, 1H, H_a), 3.85–3.50 (m, 2H, H_b), 3.25–2.95 (m, 2H, H_c). These data is fully consistent with the data reported in the literature for 2-phenyl-1,2,3,4-tetrahydroisoquinoline-1-carbonitrile (8).

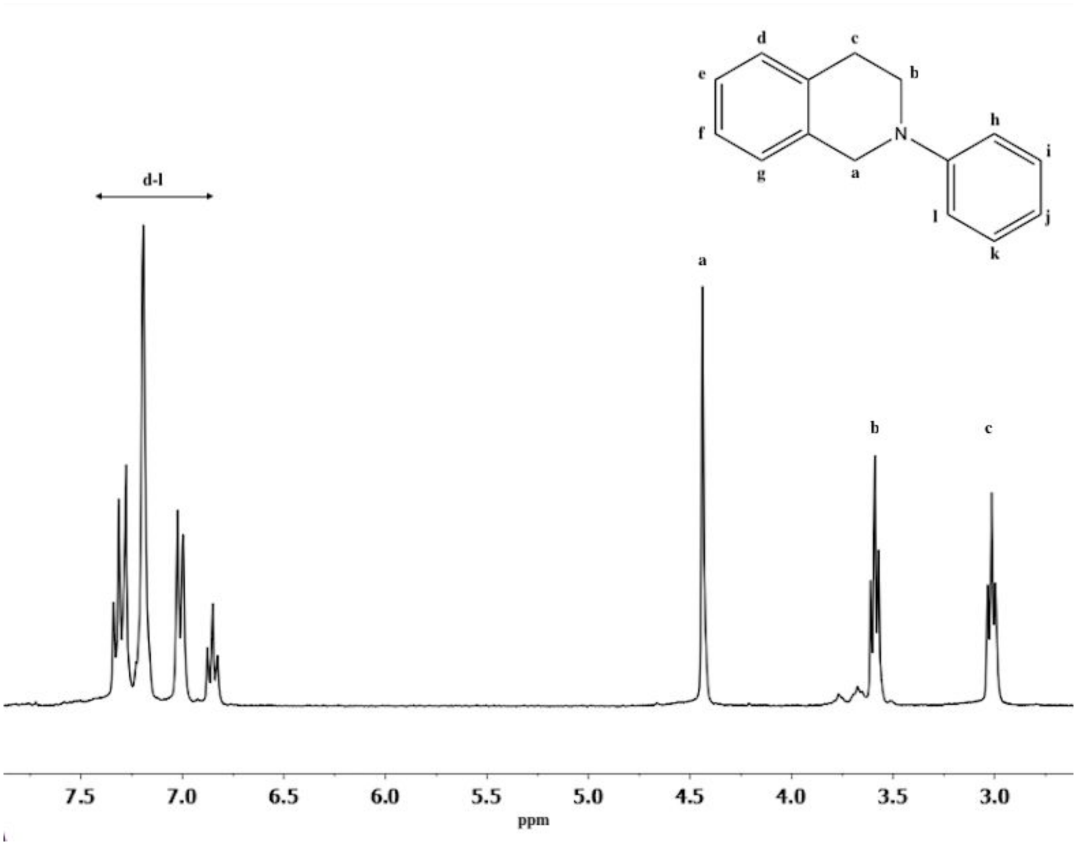

Figure S13. ^{1}H-NMR of 2-phenyl-1,2,3,4-tetrahydroisoquinoline.

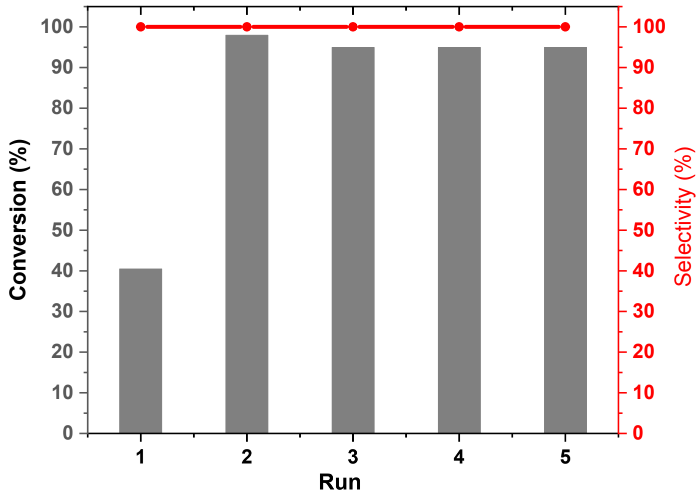

Figure S14. Recyclability of HCP-BNT2Ph in the aza-Henry reaction between 2-phenyl-1,2,3,4-tetrahydroisoquinoline and trimethylsilyl cyanide. Run 1, 4 h of irradiation, runs 2-2 h of irradiation, runs 3-5, 6h of irradiation

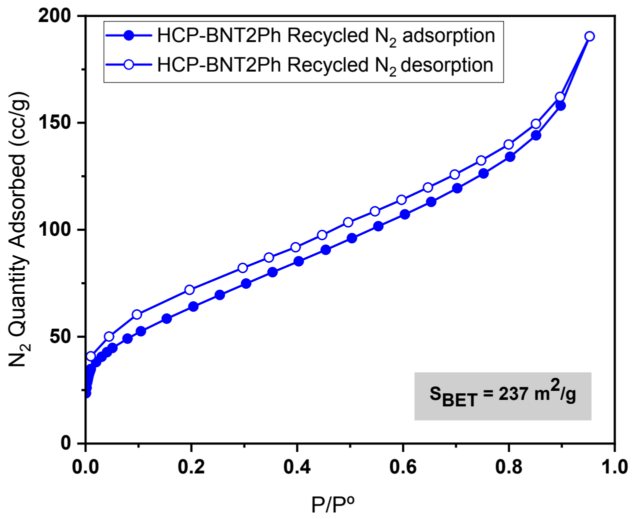

Figure S15. N_2 adsorption/desorption isotherms of HCP-BNT2Ph after five runs

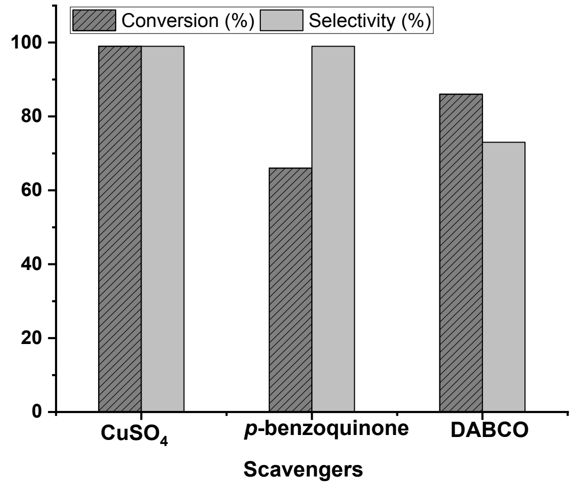

Figure S16. Scavengers tested in the aza-Henry photoreaction using HCP-BNT2Ph as photocatalyst.

Tables

Table S1. Elemental Analysis of pure HCP-BNT2Ph and after 5 consecutive uses as photocatalyst in the aza-Henry reaction

Polymer		C(%)	H(%)	N(%)
HCP-BNT2Ph	Theoretical*	89.84	5.74	2.29
HCP-BNT2Ph	Experimental	69.46	5.93	3.03
HCP-BNT2Ph (recycled)	Experimental	61.68	4.03	3.93

*Calculated for a repeat unit containing 1 BN-tetraphene for each 2 biphenyl groups

Table S2. Literature data for aza-Henry coupling using 2-phenyl-1,2,3,4-tetrahydroisoquinoline (THIQ) as substrate and nitromethane as nucleophile and metal-free porous organic polymers as photocatalyts.

Catalyst (Amount)	THIQ amount	S_{BET} (m^2/g)	Conditions	Oxidant	Time (h)	Conver. (%)	Ref
RB-CMP (2 mol%)	0.5 mmol (104.6 mg)	833	60 W bulb light	Air	12	95	9
EY-POP1 (10 mg)	0.2 mmol (41,8 mg)	587	14 W household bulb	Air	24	98	10
CPP-PhIm-2F (1 mg)	20 mg	285	15 W White light	Air	48h	85.3	11
CPP-So2-PhIm-2F (1 mol%)	20 mg	684	15 W White light	Air	48h	80	12
TPPy-PBT-COF (5 mg)	0.2 mmol (41,8 mg)	0.1861	5 W blue LED lamp	O_2 (1 bar)	10	86	13
HCP-BNT2Ph (40 mg)	1.5 mmol (313.93)	612	50 W blue LED	Air	**4 h** (1st Run) **2 h** (2nd run)	98 98	This work

References

(1) Valencia, I.; García-García, P.; Sucunza, D.; Mendicuti, F.;Vaquero, J. J. 1,10a-Dihydro-1-aza-10a-boraphenanthrene and 6a,7-Dihydro-7- aza-6a-boratetraphene: Two New Fluorescent BN-PAHs. *J. Org. Chem.* **2021**, *86*(23), 16259–16267.

(2) Fuerte-Díez, B.; Valverde-González, A.; Pintado-Sierra, M.; Díaz, U.; Sánchez, F.; Maya, E. M.; Iglesias, M. Phenyl Extended Naphthalene-Based Covalent Triazine Frameworks as Versatile Metal-Free Heterogeneous Photocatalysts. *Solar RRL*, **2022**, *6*(2), 1–10.

(3) Gaussian 16, Revision C.01; Frisch, M. J. et al. Gaussian, Inc., Wallingford CT, 2016.

(4) Zhao, Y. & Truhlar, D. G. The M06 suite of density functionals for main group thermochemistry, thermochemical kinetics, noncovalent interactions, excited states, and transition elements: Two new functionals and systematic testing of four M06-class functionals and 12 other functionals. *Theor. Chem. Acc.* **2008**, *120*, 215-241.

(5) a) Reed, E., Curtiss, L. A., Weinhold, F. Intermolecular interactions from a natural bond orbital, donor-acceptor viewpoint. *Chem. Rev.* **1988**, *88*, 899; b) Foster, J. P., Weinhold, F. Natural hybrid orbitals. *J. Am. Chem. Soc.* **1980**, *102*, 7211; c) Reed, A. E., Weinhold, F. J. Natural localized molecular orbitals. *Chem. Phys*. **1985**, 83, 1736.

(6)Glendening, E. D., Badenhoop, J. K., Reed, A. E., Carpenter, J. E., Bohmann, J. A., Morales, C. M., Weinhold, F. Theoretical Chemistry Institute, University of Wisconsin, Madison, 2001.

(7) Reed, A. E., Weinstock, R. B., Weinhols*,* F. Natural population análisis. *J. Chem. Phys.* **1985**, *83*, 735.

(8) Zhu, S.; Liu, Y.; Chen, X.; Qu, L.; Bing, Y. Polymerization-Enhanced Photocatalysis for the Functionalization of C(sp3)−H Bonds. ACS Catal. **2022**, 12, 126−134

(9) Jiang, J. X.; Li, Y.; Wu, X.; Xiao, J.; Adams, D. J.; Cooper, A. I. Conjugated Microporous Polymers with Rose Bengal Dye for Highly Efficient Heterogeneous Organo-Photocatalysis. *Macromolecules*, **2013**, *46*(22), 8779–8783.

(10) Wang, C. A.; Li, Y. W.; Cheng, X. L.; Zhang, J. P.; Han, Y. F. Eosin Y dye-based porous organic polymers for highly efficient heterogeneous photocatalytic dehydrogenative coupling reaction. *RSC Advances*, **2017**, *7*(1), 408–414.

(11) Monterde, C., Navarro, R.; Iglesias, M.; Sánchez, F. Fluorine-Phenanthroimidazole Porous Organic Polymer: Efficient Microwave Synthesis and Photocatalytic Activity. *ACS Appl. Mater. Inter.* **2019**, *11*(3), 3459–3465.

(12) Monterde, C.; Pintado-Sierra, M.; Navarro, R.; Sánchez, F.; Iglesias, M. Effective Approach toward Conjugated Porous Organic Frameworks Based on Phenanthrene Building Blocks: Metal-Free Heterogeneous Photocatalysts. *ACS Appl. Mater. Inter.* **2020**, *12*(13), 15108–15114.

(13) Yang, F.; Li, C. C.; Xu, C. C.; Kan, J. L.; Tian, B.; Qu, H. Y.; Guo, Y.; Geng, Y.; Dong, Y.B. A covalent organic framework as a photocatalyst for window ledge cross-dehydrogenative coupling reactions. *Chem. Comm.* **2022**, *58*(10), 1530–1533.
